# Convalescent Plasma Rescued a Severe COVID-19 Patient with Chronic Myeloid Leukemia Blast Crisis and Myelofibrosis

**DOI:** 10.4274/tjh.galenos.2020.2020.0400

**Published:** 2021-02-25

**Authors:** Lu-Lu Zhang, Yu Liu, Yi-Gang Guo, Juan Chang, Bo Gao, Zhang-Zhi Li, Wei Geng, Pin Hu, Bin Song, Xia Zhang, Chu-Cheng Wan

**Affiliations:** 1 Taihe Hospital Affiliated to Xi’an Jiaotong University Health Science Center, Department of Hematology, Shiyan, China; 2 Taihe Hospital Affiliated to Xi’an Jiaotong University Health Science Center, Department of Laboratory Medicine, Shiyan, China

**Keywords:** COVID-19, Chronic myeloid leukemia, Blast crisis, Convalescent plasma therapy

## To the Editor,

Coronavirus disease-2019 (COVID-19) is now an unprecedented worldwide pandemic. However, there are no specific antiviral drugs available for its treatment. A handful of studies have summarized convalescent plasma (CP) transfusion in severe or critical cases [1,2,3,4,5,6], whereas therapies for COVID-19 in cases of hematologic cancer are rather limited. We give the first report of the initial clinical experience with CP transfusion administered to a severe COVID-19 patient with chronic myeloid leukemia blast crisis (CML-BP) and myelofibrosis.

**Figure 1:**
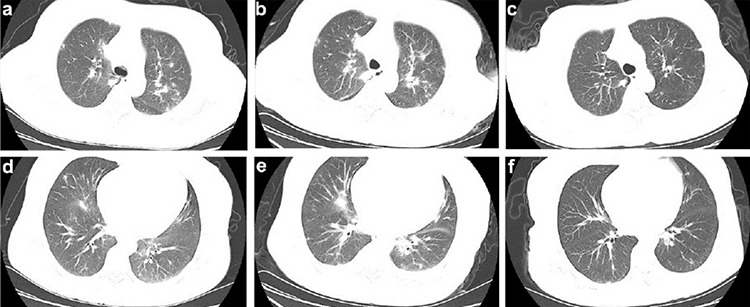
Representative chest computed tomography (CT) scans of this patient. a, d) Chest CT scan obtained at admission showed multiple ground-glass opacities with uneven density involving both upper lungs and right lung. b, e) Chest CT obtained on February 25 before CP transfusion, where multiple shadows of high density in both upper lungs and patchy consolidation in the right lung were observed. c, f) CT image taken on March 6 showed the absorption of bilateral ground-glass opacity after CP transfusion.

**Figure 2:**
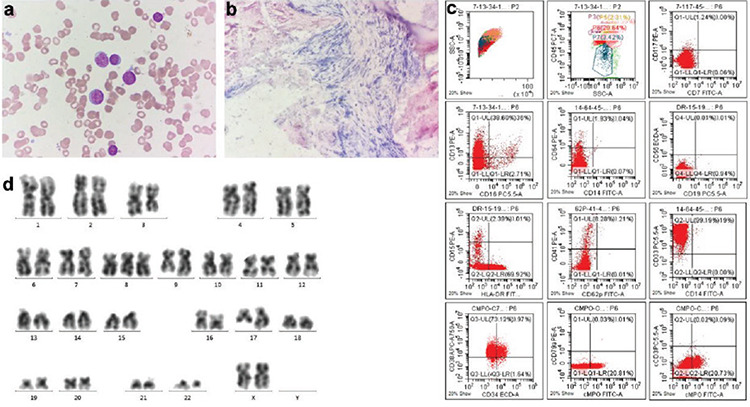
Morphology, flow cytometry, and cytogenetics of the patient at admission. a) Bone marrow aspirate smear (Wright-Giemsa stain, original magnification 1000x) showed leukemic blast cells. b) Bone marrow biopsy (H&E stain, original magnification 100x) showed fibrosis. c) Flow cytometry plots showed a single cluster of blasts with positivity for CD33 and CD13 and partial CD41, CD34, HLA-DR, and cMPO. d) Karyotype of bone marrow showed t(9;22) and t(3;17) abnormality in the same leukemic clone cells.

A 46-year-old female patient presented with diarrhea, a cough with clear sputum, and fatigue for 3 days. Her previous history of treatment for CML-BP consisted of daunorubicin at 45 mg/m2 for 3 days and cytarabine at 200 mg/m2 for 7 days in a continuous infusion, and then she experienced discontinuation of the tyrosine kinase inhibitor therapy. She was given imatinib (600 mg/day) starting in November 2017, but a drug-related hematologic adverse event occurred quickly. As a result, dasatinib (150 mg/day) was given instead. She had not achieved complete hematological remission at the diagnosis of COVID-19 due to poor responses to these therapies. At admission (February 21, 2020), the most relevant clinical findings included white blood cell count of 4.93x10^9^/L with 78% neutrophils, 9.2% lymphocytes, 2.3% basophils, 0.4% eosinophils, and 10.1% monocytes; hemoglobin of 51 g/L; platelet count of 79x10^9^/L; high-sensitivity C-reactive protein of 57.43 mg/L; and interleukin-6 level of 59.25 pg/mL. The real-time polymerase chain reaction (RT-PCR) assay of the throat swab was positive for severe acute respiratory syndrome-coronavirus-2 (SARS-CoV-2) infection. A chest CT obtained on February 21 revealed bilateral ground-glass opacities primarily distributed along the pleura (Figures 1a and 1d). Bone marrow examination and flow cytometry suggested a blast crisis, with 20.5% of leukemic blasts (Figure 2a) that expressed CD33 and CD13 and partially CD41, CD34, HLA-DR, and cMPO (Figure 2c). RT-PCR revealed a major chimeric BCR-ABL1 transcript. Fluorescence in situ hybridization analysis confirmed the BCR-ABL1 fusion rearrangement signal. Gene sequencing showed no mutation in the ABL1 kinase domain. Cytogenetics were characterized as 46,XX,t(3;17)(q21;q21),t(9;22)(q34;q11)[19]/47,idem,+8[1] (Figure 2d). A biopsy specimen detected grade 2 fibrosis (Figure 2b) according to the marrow fibrosis scoring system. Computed tomography (CT) scanning showed hepatosplenomegaly (21 cm in length). The disease was consistent with COVID-19 and CML-BP with myelofibrosis.

The patient developed worsening hypoxemia, with oxyhemoglobin saturation (SaO_2_) oscillating between 90% and 93%, after receiving conventional antiviral therapy including arbidol (200 mg three times daily), oseltamivir (75 mg twice daily), ribavirin (500 mg every 12 hours), and interferon-alpha-2b inhalation (5 million units twice daily). A follow-up chest CT scan showed increased consolidation and extended opacities (Figures 1b and 1e). On February 26, the patient received a transfusion of 200 mL of CP obtained from a donor who had recovered from SARS-CoV-2 infection in January 2020 with the neutralizing antibody titer above 1:640. No immediate adverse reactions were observed after plasma infusion. One day later, her SaO_2_ increased to 98% with oxygenation index of 200 mmHg. At the same time, her clinical symptoms and pathological criteria improved rapidly within 3 days. The patient’s condition improved to stable; thus, treatment with pulsed dasatinib was administered (100 mg once daily). Three repetitive RT-PCR tests were negative from the 6^th^ to 8^th^ day after CP transfusion. Chest images showed absorption of opacities within 10 days (Figures 1c and 1f). The patient recovered and was discharged on the 14^th^ day of admission.

A recent study showed a 10% case rate of COVID-19 among 128 patients with hematological cancers in Wuhan [7]. The treatment of severe COVID-19 has been challenging. This pilot study on CP therapy shows that it can serve as a promising rescue option for hematologic cancer patients with severe COVID-19, which warrants further investigation by randomized trials.
